# Development of Social Vocalizations in Mice

**DOI:** 10.1371/journal.pone.0017460

**Published:** 2011-03-09

**Authors:** Jasmine M. S. Grimsley, Jessica J. M. Monaghan, Jeffrey J. Wenstrup

**Affiliations:** 1 Department of Anatomy and Neurobiology, Northeastern Ohio Universities Colleges of Medicine and Pharmacy, Rootstown, Ohio, United States of America; 2 MRC Institute of Hearing Research, University Park, Nottingham, United Kingdom; University of Washington, United States of America

## Abstract

Adult mice are highly vocal animals, with both males and females vocalizing in same sex and cross sex social encounters. Mouse pups are also highly vocal, producing isolation vocalizations when they are cold or removed from the nest. This study examined patterns in the development of pup isolation vocalizations, and compared these to adult vocalizations. In three litters of CBA/CaJ mice, we recorded isolation vocalizations at ages postnatal day 5 (p5), p7, p9, p11, and p13. Adult vocalizations were obtained in a variety of social situations. Altogether, 28,384 discrete vocal signals were recorded using high-frequency-sensitive equipment and analyzed for syllable type, spectral and temporal features, and the temporal sequencing within bouts. We found that pups produced all but one of the 11 syllable types recorded from adults. The proportions of syllable types changed developmentally, but even the youngest pups produced complex syllables with frequency-time variations. When all syllable types were pooled together for analysis, changes in the peak frequency or the duration of syllables were small, although significant, from p5 through p13. However, individual syllable types showed different, large patterns of change over development, requiring analysis of each syllable type separately. Most adult syllables were substantially lower in frequency and shorter in duration. As pups aged, the complexity of vocal bouts increased, with a greater tendency to switch between syllable types. Vocal bouts from older animals, p13 and adult, had significantly more sequential structure than those from younger mice. Overall, these results demonstrate substantial changes in social vocalizations with age. Future studies are required to identify whether these changes result from developmental processes affecting the vocal tract or control of vocalization, or from vocal learning. To provide a tool for further research, we developed a MATLAB program that generates bouts of vocalizations that correspond to mice of different ages.

## Introduction

Mice are highly vocal animals, with both males and females vocalizing in same-sex and cross-sex social encounters [1], [2], [3], [4], [5]. Mouse pups are also highly vocal, producing isolation vocalizations when they are cold or removed from the nest, despite the fact that they cannot hear until postnatal day 10 (p10) [6], [7]. Adult mice can discriminate between vocalizations of pups and adults. For example, virgin female mice are attracted to playbacks of male song, but not pup vocalizations [8]. In contrast, playback of pup vocalizations to mothers, but not to pup-naïve virgins, elicits search and retrieval behavior [9], [10]. These behavioral differences have a correlate in the auditory cortex, where physiological responses to pup syllables differ for maternal and virgin animals [11], [12]. The complexity of acoustic communication behaviors and the presence of neural correlates that underlie some of these behaviors provide a strong rationale to explore the details of this vocal communication system and how it changes developmentally.

Previous work has shown that the frequency and duration of pup vocalizations change during development. Liu and colleagues [5] reported that adult syllables are shorter than pup syllables and that the peak frequency of the syllables also differs between adults and pups, with adult syllables having frequencies that fall between the two frequency ranges occupied by pup syllables. However, it is not clear that these changes occur for all of the pup syllable types characterized by Scattoni and colleagues [13]. If so, the changes might reflect maturation of the vocal tract or vocal control. If all of the syllable types do not change in the same way, it may suggest that vocal learning occurs as the acoustic features of syllables are refined. In adults, syllables are produced in bouts that have a song-like structure [14]; different syllable types are produced in temporally organized patterns. How these adult patterns develop is not known, nor is the relationship between them and the patterns of pup isolation vocalizations understood. Changes in syllable sequencing that occur during development may provide additional cues for adult animals to differentiate the calls of mouse pups from those of adults. To address these issues, we examined the structure and sequencing of syllables across development and compared these features to those in adults.

We characterized the vocalizations of CBA/CaJ mice because they are the standard *normal control* strain used for auditory research, due to their sensitive hearing thresholds that are maintained up to at least 39 weeks [15]. We recorded isolation vocalizations from pups between ages p5 and p13, and from adults in a variety of social settings. The results show that both the probability of different syllable types being produced and their sequencing within bouts changed with the age of pups, and that these features differed from adults. We used these data to generate a MATLAB program we have called a ‘virtual mouse vocal organ’. This probabilistic model of mouse vocalizations generates bouts of mouse syllables with acoustic features appropriate to mice of different ages. This program allows for highly controlled generation of vocal stimuli that correspond to those of mice at different developmental stages for use in behavioral and neurophysiological experiments.

## Materials and Methods

### Ethics Statement

All procedures were approved by the Institutional Animal Care and Use Committee at the Northeastern Ohio Universities Colleges of Medicine and Pharmacy (Approval ID number 10-001). A total of 42 CBA/CaJ mice were used in this study, comprising 18 adult males, 12 adult females (aged between p91 and p140) and 15 pups from 3 litters with different parents.

### Acoustic recordings

**Figure 1:**
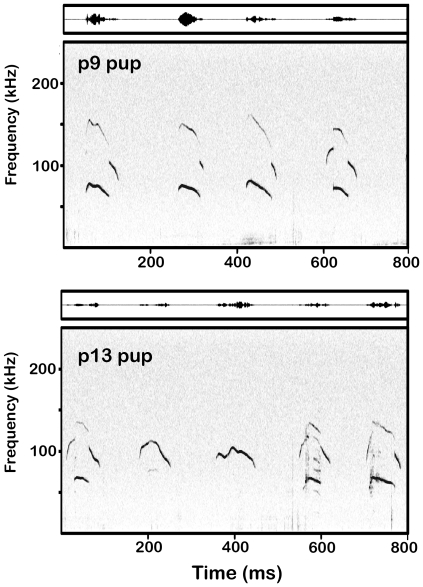
Spectrograms and waveforms of typical syllables recorded from CBA/CaJ pups. All of the syllables shown from a p9 pup (top) have frequency steps and one harmonic, the sound levels of consecutive syllables varied substantially. The final two syllables shown from a p13 pup (bottom) have nonlinearities within their 2nd elements.

Recordings of mouse vocalizations were carried out in a single-walled acoustic chamber (Industrial Acoustics, New York, NY) lined with anechoic foam. Mice were situated within an open topped cylindrical arena (D 165 mm, H 110 mm) placed over a heating pad. Acoustic signals were recorded by ultrasonic condenser microphone (CM16/CMPA, Avisoft Bioacoustics, Berlin, Germany), located 5 cm (for pups) or 7 cm (for adults) above the arena floor. The acoustic signals were amplified and then digitized at 500 kHz with 16-bit resolution (UltraSoundGate, Avisoft Bioacoustics). Recorded signals were displayed in real-time on a computer with commercial software (Recorder_USGH, Avisoft Bioacoustics). Gain was adjusted online to prevent signal saturation. The recording system was flat (±3 dB) from 20 kHz to 140 kHz, with a low frequency roll-off of 12 dB per octave. The system provided a strong signal-to-noise ratio that did not require offline filtering (see unfiltered recording sequence in Figure 1).

Recordings of pup isolation vocalizations, lasting 5 minutes, began 5–10 seconds prior to placing the pup in the center of the arena. Pup vocalizations were recorded from 15 pups at p5, and from 14 pups at the subsequent ages p7, p9, p11 and p13. Pups were from 3 litters with different parents. Mouse pups did not produce isolation vocalizations after p13, in agreement with previous work [9]. For recording adult vocalizations, several behavioral paradigms were used: male-male interactions (4 pairs), male-female interactions (6 pairs), female-female interactions (3 pairs) and male vocalizations in the presence of female bedding (4 animals). In adult encounters, one animal was introduced to the recording chamber a few seconds prior to the second animal. Recording was started when the first animal was introduced and continued for 30–60 minutes.

### Data Analysis

#### Basic analyses of spectro-temporal features

Syllables were detected offline using SASLab Pro 5.1 (Avisoft Bioacoustics). Since syllable amplitude was variable (Fig. 1) and sometimes close to the background noise floor, automated thresholding was not used to detect sounds. Instead, the start and end times of the syllables were manually tagged onto the sound file, providing a measure of syllable duration and marking syllables for further analysis. The dominant frequency, the frequency that was produced at the maximum amplitude, was also measured at several points within a syllable: the start (+2 ms), center and end (−2 ms). All syllables recorded were included in the analysis so long as they were not saturated or associated with movement noise.

Syllables were separated into distinct categories based on their spectro-temporal characteristics. The classification scheme used by Scattoni *et al.*, (2008) provided an initial guide, and in most cases we used their syllable categories. Several frequency metrics were calculated depending on the spectro-temporal characteristics of the syllable type. The dominant frequency was computed for constant frequency syllables (the flat syllable and the short syllable) and syllables that had both upward and downward frequency modulations (the complex syllable and the chevron syllable). The bandwidth of frequency modulated syllables was calculated by subtracting the dominant frequency at the lowest frequency of the syllable from the dominant frequency at the highest frequency of the syllable. For syllables with frequency steps, the dominant frequency of each ‘component’ was computed along with the frequency difference between components, i.e., the step size. These characteristics were compared among pups of different ages and between each pup age group and adults.

#### Zipf relation

Zipf's law was developed to describe the proportion of word usage within human language; it holds that within a natural language, the frequency of occurrence of a word is inversely proportional to its rank in the frequency table [16], [17]. We compared the Zipf's relation value for the vocal repertoire in order to identify whether the use of each syllable type was non-random and to calculate the capacity for the mouse vocal repertoire to carry information. The Zipf's relation was compared among pup groups and adults. Slopes of the Zipf relation were computed as the regression coefficient when the logarithm of frequency of occurrence of each syllable is plotted against the logarithm of the syllable rank (the most common syllable, the second most common syllable and so on) [16], [17]. The resulting slope was used as a measure of the potential of the repertoire to carry information from the caller to the listener. A slope of −1 is considered to represent the optimal balance between the number of syllable types in an animal's vocal repertoire and the level of repetition of each syllable type [18]. A shallower, slope (closer to 0) represents a repertoire that is more diverse and random, whereas a steeper, more negative, slope represents a repertoire that is less diverse and more repetitious.

### Syllable sequence analysis

The Zipf's statistic provides a measure of the potential for the vocal repertoire as a whole to carry information, but it does not reveal higher-order structure that may be present in sequences of syllables within bouts.

We used two statistical methods to test whether successive syllables within a syllable bout were independent of one another, or conversely, whether there was an effect caused by one or more of the preceding syllables in the bout. We used both a chi-squared goodness-of-fit test and a higher-order entropy model based on information theory. Chatfield and Lemon [19] outlined the advantages of using both methods in unison. The chi-squared test determines whether the repertoire is random or has some higher-order structure. However, it does not clearly determine the sequential level at which the repertoire has higher-order structure. Information theory entropy analysis provides a graphical representation of the data that allows for comparisons between the levels of organization, information entropy, of the repertoire at higher-orders. The information theory entropy measure also allows for comparison between syllable repertoires from different groups of animals.

We used the chi-squared goodness-of-fit test at the one-syllable and the two-syllable levels to test whether the repertoire had internal structure. At the one-syllable level, the proportions of each syllable type were compared against a random model using a chi-squared analysis, testing whether the syllables were produced in a nonrandom pattern. At the two-syllable level, we examined syllable combinations for all bouts having more than 3 syllables, at all ages. The frequency of occurrence of each possible two-syllable type pairing was compared, using chi-squared, to a random model. A significant difference reflects an ordered structure within the repertoire where the probability of a particular syllable being produced is affected by the previous syllable; however it does not provide specific information as to what the probable patterns are. We further used the two-syllable pairings to assess the complexity of the repertoire across development. We considered a repertoire to be more complex if there was a higher probability of switching between syllable types than repeating the same syllable type. This was achieved by summing all repetitions, regardless of syllable type, and dividing the sum by the total two-syllable pairings.

We used information theory to measure the entropy of the repertoire at several different levels; zeroth order, first order, second order and third order. Zeroth order measures the diversity of the vocal repertoire, in this case how many syllable types there are at each age. First-order entropy measures the simple organizational structure of the repertoire, how often each syllable type is used; this is akin to the Zipf's statistic. Second-order entropy measures the level of organization at the two-syllable sequence level. Third-order entropy describes the extent to which the bouts are organized at the three-syllable level. The highest level we compared is fourth-order entropy; this investigates to what extent the bouts are organized at the four-syllable level. If a repertoire has organization at higher levels, then the entropy should reduce significantly with each increasing level. We used equations for the formation of informational entropies described by Doyle *et al.* [20]:
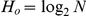
where H_0_ is the maximum entropy of the vocal repertoire in bits, the zeroth-order entropy, and *N* is the total number of syllable types. First-order entropy is calculated using:
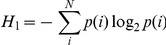
where *p(i)* is the probability of a syllable type being produced within the mouse vocal repertoire at a given age. Second-order entropy is reduced by any higher-order structure present within the repertoire:
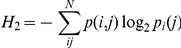
where *p(i,j)* is the joint probability of two syllable types being produced and *P_i_(j)* is the conditional probability that syllable *j* will be produced given that syllable *i* has just occurred within a bout. Third-order entropy goes on to measure the entropy, in bits, at the three-syllable level:
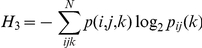
where *p(i, j, k)* is the combined probability of three syllable types being produced and *P_ij_(k)* is the conditional probability that *k* will be produced given that both *i* and *j* have just been produced sequentially.

When the entropy value of the repertoire, in bits, is plotted against the entropic level, zeroth to fourth order, the degree of change between two entropic orders reflects the level to which the repertoire is reliant on higher-order sequential patterns. In a truly random system the entropic value would be equal at all levels. A significant reduction in entropy between orders is indicative of a pattern in the sequencing of syllables within bouts, at least up to that level. It has been proposed that vocal learning animals learn the higher-order structure of their communication system, so that the adult repertoire would have a greater level of higher-order structure than that of pups [18]. We compared the differences between the entropy at different levels using a chi-squared analysis. For example, comparison of the change in entropy between 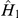 and 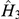
was made using the following [19]:
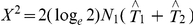
where N_1_ is the total number of syllables sampled and 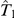 is 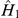-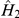
and 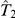 is 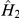-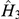. This can be used to compare differences between values at each entropic level, where 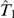 would be the difference between 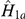 and 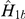, where *a* and *b* are the repertoires from animals of different ages. This measure gives the diversity of the proportion of each syllable used in the repertoire, which is akin to comparing the Zipf's statistic ratios across ages. For this analysis, the vocal repertoire should comprise similar numbers of syllable types, as the T values would be expected to be greater when there are more syllable types. Large samples are needed for these analyses because the degrees of freedom become very large at the higher entropic levels. The degrees of freedom are calculated as described by Chatfiel and Lemon [19] (see Table 1). Where c is the number of syllable types produced. When testing for a significant difference in the level of entropy between two non-adjacent entropy levels, for example from 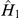 and 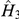, the degrees of freedom are the sum of those at 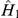 and 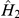: (DF = (*c*-1)^2^+*c*(*c*-1)^2^). If there is a significant decrease in entropy between 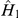 and 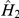, but not between 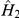 and 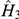, it shows that the communication system has internal structure up to the two-syllable level, but no additional structure at the three-syllable level. We compared the magnitude of the change, across age, between 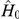 and 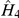. This measured the difference in the level of higher-order structure across ages, providing a measure of the age-related changes in the organization of the bouts at the two-syllable level (the change in entropy between 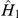 and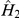) up to the four-syllable level (the change in entropy between 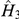
and 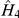). If the change is increasingly negative as pup development proceeds, there is an increasing level of higher-order structure in the sequencing of syllables.

**Table 1:** Equations for calculating the degrees of freedom at each entopic level.

Entropic level	Degrees of freedom
Zeroth	*c*-1
First	(*c*-1)^2^
Second	*c*(*c*-1)^2^
Third	*c^2^*(*c*-1)^2^

### The virtual mouse vocal organ

We generated a ‘virtual mouse vocal organ’ within MATLAB. This program generates bouts of mouse vocalizations with acoustic features that are appropriate to each of the mouse ages studied. First, a version of each syllable type which was typical in its spectro-temporal characteristics was selected for each age. Sequences of syllables are generated using a probabilistic Markov model. We examined syllable combinations at all ages for all bouts with more than 3 syllables. The probability of occurrence of each possible two-syllable type pairing was measured, as was the probability of occurrence of each three-syllable sequence. The probability of each syllable occurring first within a bout was also computed at each age.

The transitional probabilities of the model were calculated individually for each age group from the proportion of syllable pairs and triplets. For each age group either a first, second, or third-order model can be selected.

Initially, the first-order Markov model pseudo-randomly selects from the syllable types on the basis of their probability of occurrence. Thus the most probable first syllable will not always be selected. The second syllable is selected in the same way, using the transitional probabilities of each syllable being vocalized following the syllable type that was selected for syllable one. The third syllable is selected based on the probability of following the second syllable; this is independent of the first syllable. In this way an infinite number of syllables can be produced, and the selection of each syllable added to the syllable sequence is affected by the probability with which it follows the previous syllable. Within the second-order Markov model, each new syllable is selected pseudo-randomly based on the associated probability that it follows the preceding two syllables. The program outputs wav files made up of the typical syllables arranged in a probable sequential pattern that is appropriate for each age from p5 to p13 and for adults. The bouts are generated with the associated average inter-syllable intervals.

## Results

Although syllables were occasionally produced in discrete utterances, both pups and adults generally produced syllables in bouts that comprised several syllables. A large number of syllables were collected and analyzed from animals at each age; p5, n = 3145; p7, n = 4329; p9, n = 6306; p11, n = 4560; p13, n = 3082; adults, n = 6963 (male-male, n = 1382, male-female, n = 4121, female-female, n = 188, male with female bedding, n = 1272).

We first discuss the prevalence of different syllable types across pup age and then compare the spectro-temporal characteristics of the syllables. Finally, we characterize the age-related changes in the sequencing of the syllable bouts.

### Vocal repertoire

#### Syllable types

**Figure 2:**
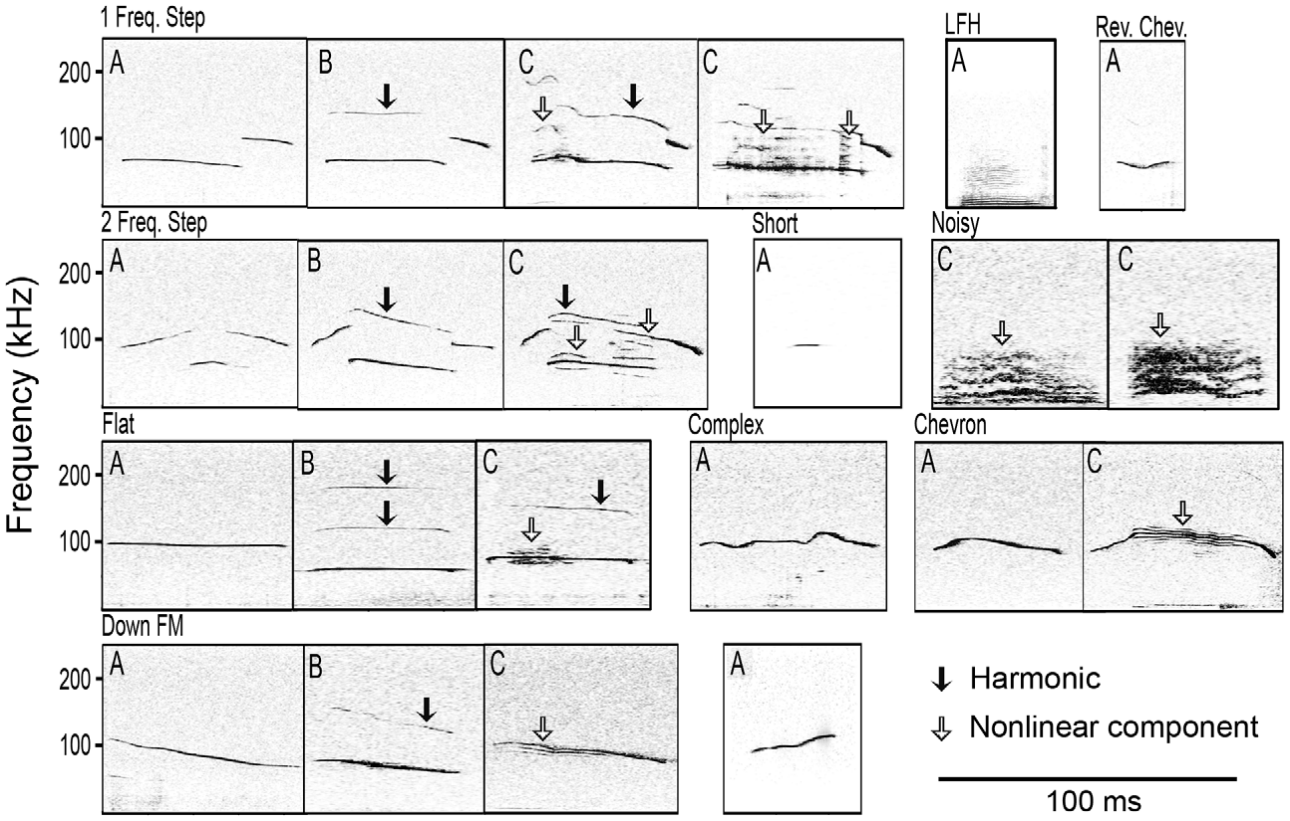
Spectrograms of the different syllable types produced by CBA/CaJ mice. The majority of syllables have energy solely in the ultrasonic range (20 kHz) with the exception of the Noisy syllable and the low frequency harmonic (LFH) syllable. Note substantial energy at frequencies above 100 kHz for many syllables. Tonal syllables are marked (A); these syllables have no harmonics or nonlinearities. Harmonic sounds are marked with a (B). Nonlinear sounds are marked with a (C); these syllables have subharmonics or deterministic chaotic elements.

We identified 11 syllable types, 9 of which were similar to those described by Scattoni *et al.*, [13]. In contrast to their study, however, we did not classify harmonic (termed composite by Scattoni *et al.*, [13]) or nonlinear (termed harmonic by Scattoni *et al.*, [13]) sounds as separate syllable types. Instead, within each syllable type, we noted whether a syllable was tonal, harmonic or nonlinear; these features are described in a later section. Holy and Guo [14] characterized syllables as having either frequency jumps or a sinusoidal structure; they did not break down the sinusoidal syllable type into subtypes based on the direction of frequency change, for example an upward frequency modulation or a downward frequency modulation. The syllable types we found are described below and are shown in Figure 2.

1. *Complex syllables* were monosyllabic with two or more directional changes in frequency >6 kHz.
2. *1 Frequency step syllables* (1 freq. step) had two elements, in which the second element was ≥10 kHz different from the preceding element and there was no separation in time between steps (these are similar to the *two syllable* calls described by Scattoni *et al.*, [13] and the single frequency jumped syllables described by Holy and Guo [14]).
3. *2 Frequency step syllables* (2 freq. step) have three elements, in which the second element was ≥10 kHz different from the first and the third element was ≥10 kHz different from the second. There was no separation in time between elements (similar to the *frequency steps* syllable described by Scattoni *et al.*, [13] and the multiple frequency jumped syllables described by Holy and Guo [14]).
4. *Up-FM syllables* were upwardly frequency modulated with a frequency change ≥6 kHz.
5. *Down-FM syllables* were downwardly frequency modulated with a frequency change ≥6 kHz.
6. *Flat syllables* were constant frequency syllables with modulation <6 kHz.
7. *Short syllables* lasted ≤5 ms.
8. *Chevron syllables* were shaped like an inverted U. The highest frequency was at least 6 kHz greater than the starting and ending frequencies.
9. *Reverse Chevron syllables* were shaped like a U. The lowest frequency was at least 6 kHz less than the starting and ending frequencies.
10. *Low Frequency Harmonic syllables* (LFH) were harmonic stacks with fundamental frequencies below 5 KHz. These syllables often had harmonics extending into the ultrasonic range in adults (>20 kHz). These have previously been classified in pups as wriggling calls [21] and in adults as pain sounds [22] or low frequency harmonics [23]. These syllables were not analyzed for developmental differences because they occur at frequencies within the sharp low-frequency cutoff of the recording microphone.
11. *Noisy syllables* were warbled, noisy, harmonic syllables in the 10–120 kHz range (these are different from the low frequency harmonic syllables given by mice).

**Figure 3:**
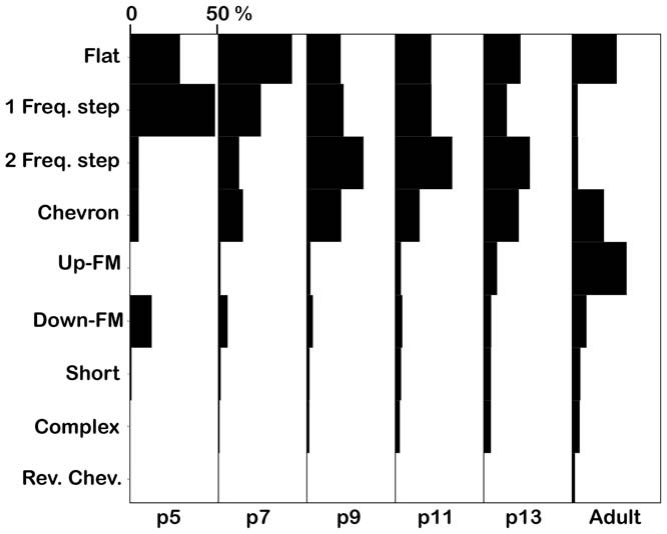
Changes in the proportions of syllable types across postnatal development. Each column shows proportions of most common syllables at the specified age. The proportions of the noisy syllable are not shown as it was only produced very rarely by adult mice, representing only 0.6% (44/6936) of syllables.

In pups of any age or in adults, some syllable types were consistently produced more commonly than other types (Fig. 3) (chi-square range 1880–8434, p<0.001). Among pups, the most commonly produced syllables were the 1 freq. step syllable (p5), the flat syllable (p7), and the 2 freq. step syllable (p9, p11, and p13). Adult mice most commonly produced the up-FM syllable. Only one syllable type was unique to adult animals, the noisy syllable.

Across age, the numbers and proportions of different syllable types changed (Fig. 3). To analyze this, we performed chi-squared analyses on scaled proportional data for the 6 most common syllable types; flat, 1 freq. step, 2 freq. step, chevron, down-FM and up-FM. There was a highly significant effect of age on the proportions of each of these major syllable types. For example, the proportion of the 2 freq. step syllable increased steadily as pups aged (Fig 3); this syllable was relatively rare among p5 syllables but was the most common syllable type by p13. The proportion of 1 freq. step syllables changed with age (Χ^2^(5, *N* = 5436) = 2475, *p*<0.001); this syllable was the most common at p5 but was rarely produced by adult animals. The most common adult syllable was the up-FM (see Fig. 2). This syllable occurred with differing proportions in different pup ages (Χ^2^(4, *N* = 562) = 383, *p*<0.001)], becoming increasingly common with age. There were also significant age-related changes in the probabilities of the flat syllable (Χ^2^(5, *N* = 7670) = 1087, *p*<0.001), the chevron syllable (Χ^2^(5, *N* = 4077) = 345, *p*<0.001) and the down-FM syllable (Χ^2^(5, *N* = 1603) = 342, *p*<0.001).

#### Complexity of vocal repertoire

**Figure 4:**
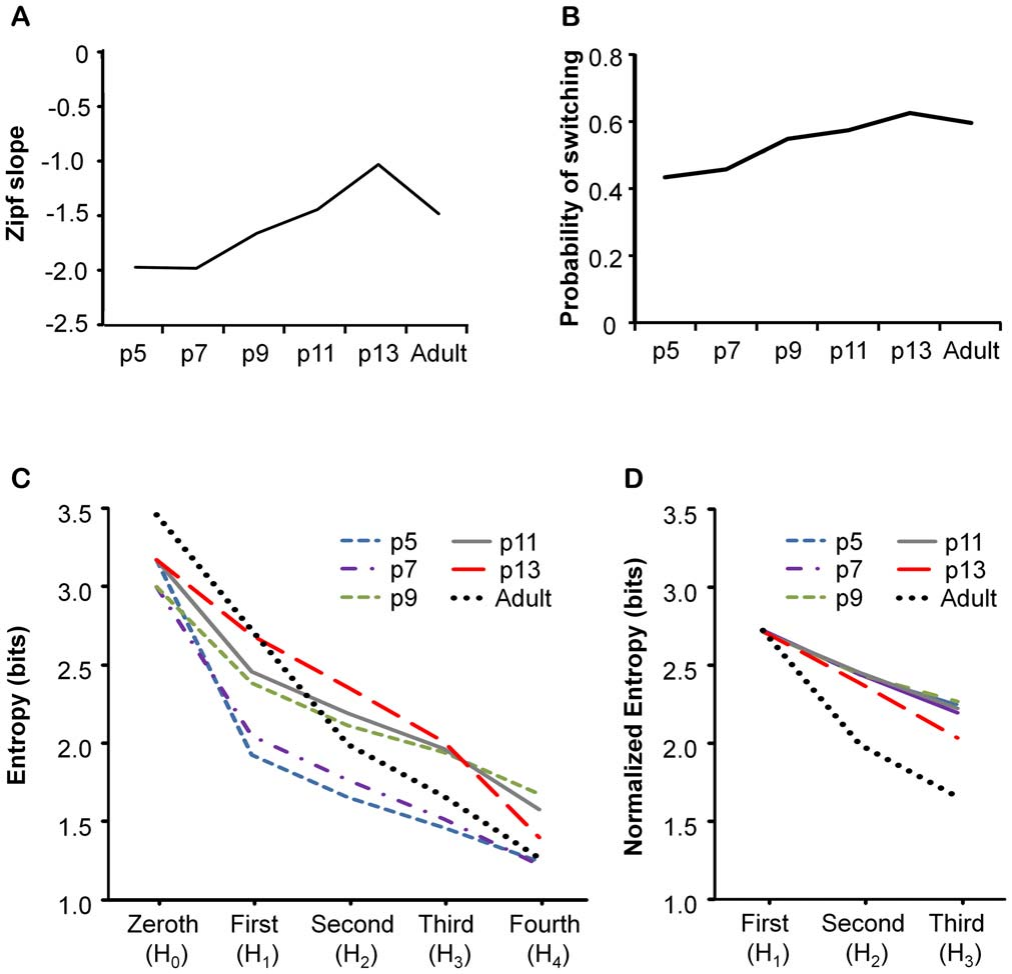
Change in the sequencing of syllables within bouts, across age. (A) The Zipf slopes increase with age, showing that the repertoire became less repetitious with increasing age. (B) The probability of a switch between syllables, in consecutive calls, increased with age. (C) For mice of different age groups, entropy declines as function of structural order. The negative slope indicates a higher-order structure in the syllable sequences at each age. (D) Slopes of the first to third order entropies, normalized to the value for adults for graphical purposes only. Note that the negative slope is steeper for p13 pups than for the younger pups, indicating greater higher-order structure within song bouts. P13 animals had significantly more sequential structure than younger animals. Adults had significantly more sequential structure than pups at any age.

A Zipf's statistic was used to compare the structural complexity of the mouse repertoire across ages. As pups increased in age, the Zipf's statistic slope became closer to −1, meaning that the second most common syllable is used half as frequently as the most common syllable, and so on (see Fig. 4A). At p5, the slope was near −2, indicating that the vocal repertoire was highly repetitious (Zipf slopes by age: p5, −1.97; p7, −1.98; p9, −1.66; p11, −1.44; p13, 1.03 and adult-1.48). By age p13, the slope was very close to the optimal −1. This value was even greater than that found from adult mouse communication syllables.

The change in entropy between H_0_ and H_1_ increased steadily from p5 to p13, indicating that the repertoire became increasingly diverse (See Fig. 4C). The entropic change between H_0_ (zero order) and H_1_ (first order) was 1.24 bits at p5 and 0.48 bits at p13 (Χ^2^ (8) = 6568, p<0.001). This change reflects the changes in Zipf's statistic that suggest that the repertoire became more diverse and less repetitious over pup development.

### Acoustic Features of Syllables

**Figure 5:**
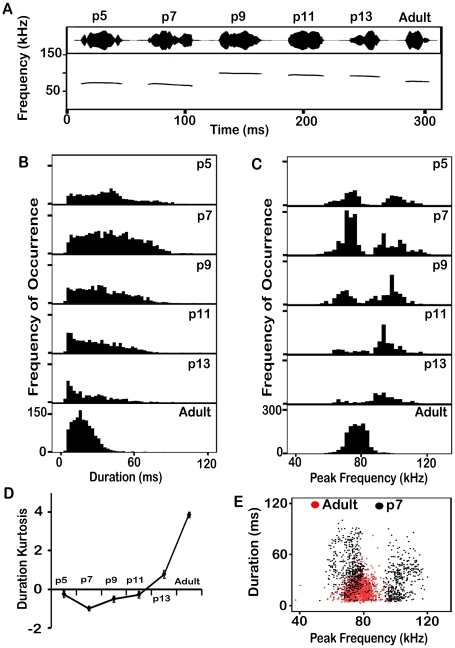
Analysis of the duration and frequency of flat syllables across ages. (A) The most typical flat syllable at each age. (B) Distributions of durations of the flat syllable at each age. Older pups are progressively less likely to produce longer flat syllables. (C) Distributions of the dominant frequency of the flat syllables at each age. At p5, p7 and p9 there are two clear peaks in the distribution, but by p11 there are fewer low frequency syllables. The frequency of the higher peak gradually reduces with age. (D) The kurtosis of the duration distribution gets more positive with age, indicating a more peaked distribution of duration. Bars represent the standard error of the mean. (E) Scatter plots of duration against frequency for flat syllables from animals aged p7 and adults. Although there is some overlap, the frequencies of adult syllables fall between those of the pup syllables.

**Figure 6:**
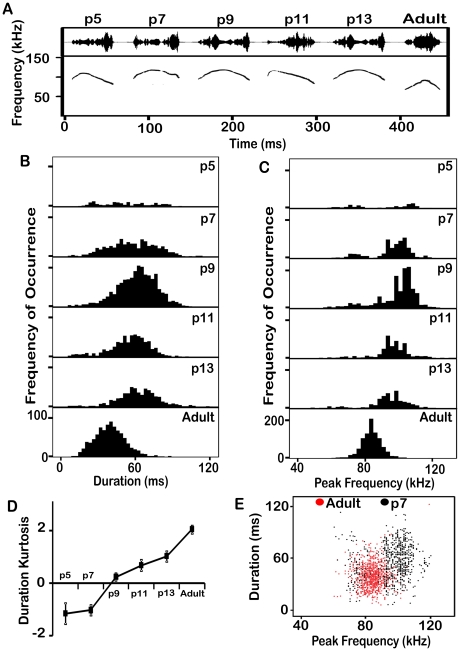
Analysis of the duration and frequency of chevron syllables across ages. (A) The most typical chevron syllable at each age. Note the similarity of pup syllables. (B) Distributions of durations of the chevron syllable at each age. The mean duration changes only slightly. (D) The Kurtosis of the distribution increases with age. Bars represent the standard error of the mean. (C) Distributions of the dominant frequency of the chevron syllable at each age. At p5 and p7 there are two clear peaks in the distribution, but by p9 there are fewer low frequency syllables being produced. The frequency of the higher peak gradually reduces with age. (E) Scatter plots of duration against frequency from animals aged p7 and adults. Although there is some overlap, the spectro-temporal features of adult syllables are distinct from pup syllables.

The acoustic features of most pup syllables changed during development, and almost always differed from the syllables of adults. The nature of these changes was complex. We illustrate developmental changes for two syllables that were common both in pups and adults, the flat syllable (Fig. 5) and the chevron syllable (Fig. 6), then follow with quantitative and statistical comparisons across age for the remaining syllables. In these analyses, all adult syllables of the same type are grouped together, since we found no significant differences in acoustic features of these syllables as a function of the category of social interaction.

In adults, the typical flat syllable had a peak frequency near 76 kHz and lasted for 19 ms (Fig. 5A). Pup syllables were either higher or lower in peak frequency, and usually longer in duration. The developmental change in duration was straightforward; younger pups produced syllables with greater variation in duration (Fig. 5A and 5D), but the proportions of longer syllables diminished as pups aged. The kurtosis of the distribution became more positive with age (Fig. 5D), showing that the mice are honing in on a more typical duration. The peak frequency of pups' flat syllables was bimodally distributed, with the lower peak just below the adult peak and the higher peak substantially higher than in adults (Fig. 5C). Over pup development, the average peak frequency increased as more syllables were produced in the higher frequency band. Although there is substantial overlap in both duration and peak frequency between pup syllables and those of adults, duration vs frequency distributions reveal much less overlap. Thus, the flat syllables of p7 pups have a distinctive distribution compared to adults (Fig. 5E).

Chevron syllables in adult mice typically had peak frequencies near 83 kHz and lasted about 40 ms. Most pup syllables were higher in frequency and longer in duration (Fig. 6). The predominant change in syllable duration over pup development was a decrease in the variance across syllables that is reflected in the change in the kurtosis of the distribution (Fig. 6D), and a slight increase in the mean (Fig. 6B). Adult versions of the chevron syllable were dramatically lower frequency than pup versions. Although the peak frequency of pup syllables was usually higher than in adults, chevron syllables in young pups, like flat syllables in pups, were bimodally distributed. As pups increased in age, most syllables had frequencies within the higher band and the average value of this band reduced (Fig. 6C). Adult chevron syllables had a unimodal distribution with less variation. Adult and pup syllables are distinct from one another in duration and dominant frequency (Fig. 6E). Both pup and adult chevron syllables had similar bandwidth.

#### Syllable duration

**Figure 7:**
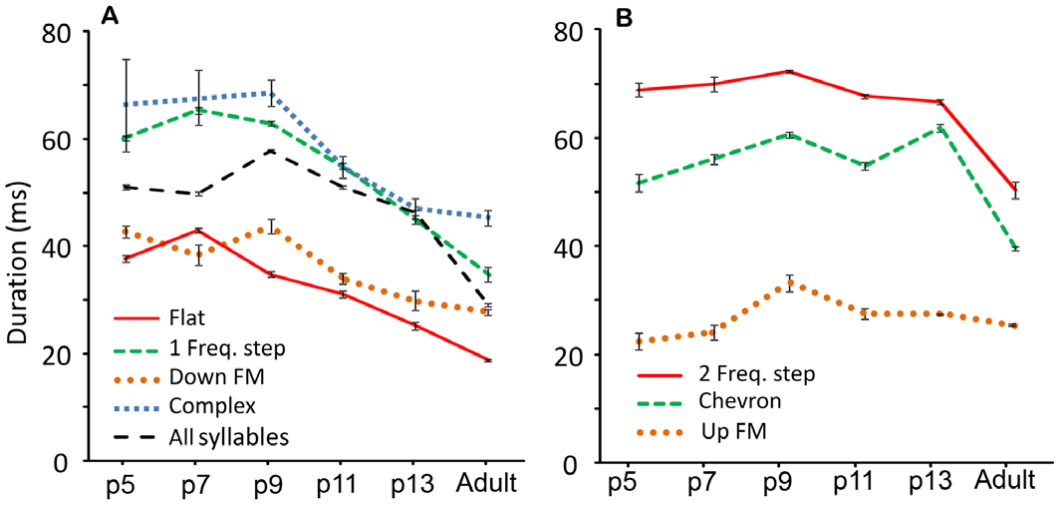
Developmental changes in syllable duration. The means and standard errors of the durations are shown. (A) These syllables showed a progressive decrease in duration over development. The average durations across all syllables (*dashed black line*) reflect the pattern of these individual syllable types. (B) These syllables had more complex patterns of duration change across age. Age-dependent durations of the short syllable are not shown, as the criterion for classifying it was duration dependent.

Overall, pup syllables were significantly longer than adult syllables (pups, 52 ms [SD 23]; adults, 29 ms [SD 20]; F (1, 28384) = 5188, p<0.001). In Figure 7, we compare the duration of each syllable type across ages. There were age-related differences in the durations of all syllable types apart from the short syllable (not shown). Most syllable types were generally shorter with age: the flat syllable (Fig. 5) (F (5, 7664) = 367, p<0.001), the 1 freq. step syllable (F (5, 5430) = 166, p<0.001), the 2 freq. step syllable (F (5, 4810) = 102, p<0.001) the down-FM syllable (F (5, 1597) = 38, p<0.001) and the complex syllable (F (5, 570) = 19, p<0.001). A common pattern was an initial increase in syllable duration between p5 and p7 or p9, followed by a decrease as pups aged further (Fig. 7A). Flat, 1- and 2-freq. step, up-FM, and complex syllables followed this pattern. The durations of the low frequency harmonic and reverse chevron syllables were not compared because too few examples were recorded from pups.

It is noteworthy that some syllables did not follow the trend described above (Fig. 7B). For example, the duration of the chevron syllable increased significantly at each age (Fig. 6B) (p<0.001), with the exception of syllables from p11 animals. Chevron syllables from p13 animals were significantly longer than those from all other pup ages (mean = 61.9 ms, 95% CI [60.5, 63.3]) p<0.001). Further, while the variability in duration often decreased with pup age, that was not always the case (e.g., complex syllables).

#### Syllable frequency

**Figure 8:**
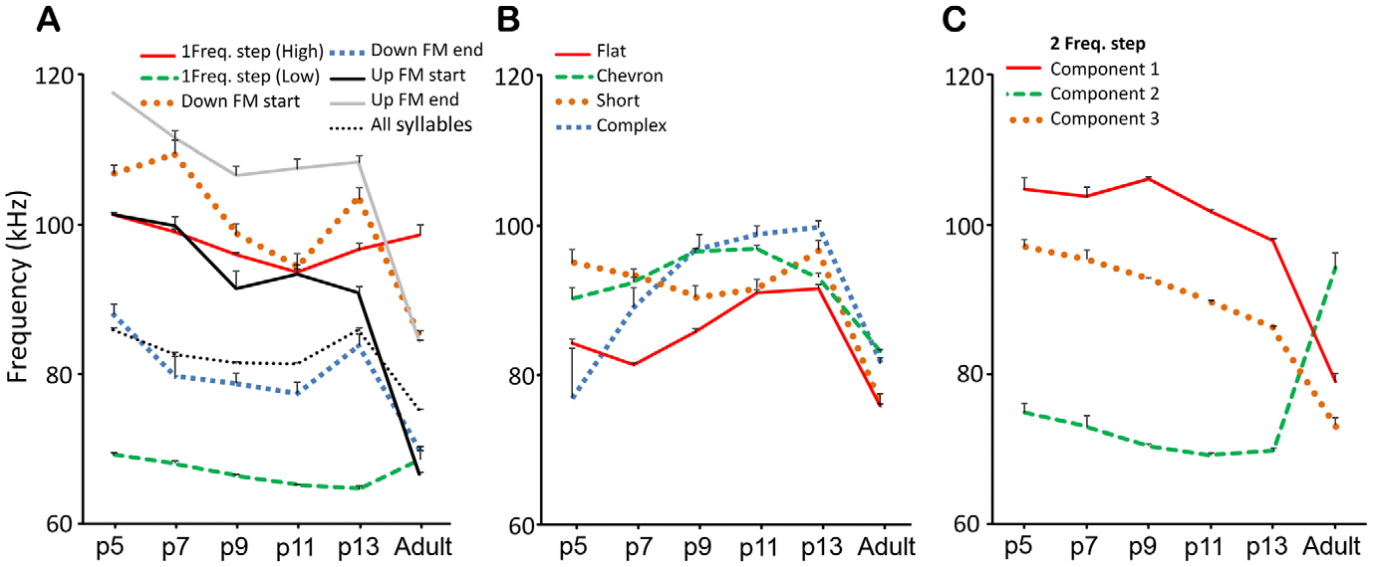
Changes in frequencies for each of several syllables, and for all syllables (*dotted black line*). (A) Dominant frequencies declined somewhat with pup age. (B) Syllable types that showed an increase in dominant frequency with pup age. In all cases, dominant frequency declined between p13 pups and adults. (C) The dominant frequencies of the three components of the 2 freq. step syllable; the stepped pattern was reversed for adult animals.

Several spectral features of syllables changed between pups and adults. On average, pup syllables were significantly higher in frequency than adult syllables (F (5, 28385) = 261, p<0.001). Moreover, when all syllables were pooled, there was an overall reduction in syllable frequency with age with the exception of p13, where there was a jump back to a frequency similar to those of p5 animals (Fig. 8A).

Although each syllable type exhibited age-related differences in spectral features, these changes were often not consistent with the general pattern (Fig. 8B). For instance, while the stepped syllables generally decreased in frequency with pup age, the peak frequency in flat and complex syllables increased. The peak frequency of the chevron syllable increased and then decreased by age p13. These observations suggest that no single mechanism is responsible for changes in syllable frequency, and they emphasize the importance of analyzing each syllable type separately.

Further inspection revealed the limitations of focusing on a single population measure (e.g., the mean peak frequency) to characterize age-related changes. Thus, Figure 5C shows that the peak frequency of flat syllables had a bimodal distribution, with a lower frequency peak at 75 kHz and a higher peak at 95 kHz. Developmental changes in the mean value represented a shift in the population from one to the other of the modes. Thus, the p5 distribution is well balanced, whereas at p7 the lower frequency syllables are more common. Conversely, at ages p9, p11, and p13, the higher frequency syllables are more common. These age-related differences are reflected in the *median* dominant frequency at each age (p5 = 73.2; p7 = 70.2; p9 = 98.6; p11 = 93.7; p13 = 91.7 and adult = 77.6).

Like the flat syllable, the chevron syllable showed a bimodal distribution of peak frequency, particularly at ages p5 and p7 (Fig. 6). At p5 there were peaks near 70 kHz and 100 kHz, with a larger proportion of higher frequency syllables (mean = 90.1 kHz; median = 108.3 kHz). By p7 there were very few syllables in the lower frequencies (mean = 92.4 kHz; median = 103.0 kHz). From ages p9 onward, the decrease in peak frequency was the result of a decrease in the mean value of the higher frequency distribution (F (5, 4071) = 106; p<0.001). Bonferoni post-hoc tests showed that the mean dominant frequencies of chevron syllables from adult animals were significantly lower in frequency than pup syllables by at least 10 kHz (mean = 83.2 kHz, 95% CI [82.7, 83.6], p<0.001). These differing patterns suggest a refinement of vocal frequency rather than modifications of the vocal tract.

The frequency step size between the two components of the 1 freq. step syllable did not change as a function of age (mean = 30.4 kHz, 95% CI [30.0, 30.8]). Within pups the dominant frequency of the lower frequency component reduced significantly as a function of age (Fig. 8A) (F (5, 5259) = 47; p<0.001). In adult mice this component had similar frequencies to younger pups. A similar pattern of frequency change was observed in the high frequency component (Fig. 8A).

The most striking difference in the 2 freq. step syllable is that the direction of the frequency step differs between pups and adults (Fig. 8C). In pups the syllable starts at high frequency, steps to a lower frequency and then steps back up to higher frequency; the adult version starts at a low frequency, steps to a higher frequency then steps back to a lower frequency. Within pups, each component of the 2 freq. step syllable reduced steadily in dominant frequency with age (fig. 8C). The first frequency step, between component 1 and component 2, was of similar magnitude amongst pups (roughly +31 kHz). However, within the adult version the step was downward and significantly smaller (mean = −15.1 kHz, 95% CI [−19.7, −10.4], p<0.001). The second frequency step was of similar magnitude amongst pups and adults.

Spectral analysis was not undertaken on the up-FM syllable at p5 as it was rarely produced at that age. Both the start end the end frequency of the up-FM syllable changed with age, with adult syllables starting at significantly lower frequencies than pup syllables (F (5, 1895) = 397, p<0.001). The upper frequencies of the up-FM syllables were significantly lower in adult animals than any pup age (M = 84.4 kHz, 95% CI [84.1, 84.7], p<0.001). The bandwidth of these syllables did not change as a function of age. The same pattern was observed for the down-FM syllable; adult syllables started and ended at lower frequencies than pup syllables and there was no overall change in the bandwidth (Fig. 8A).

**Figure 9:**
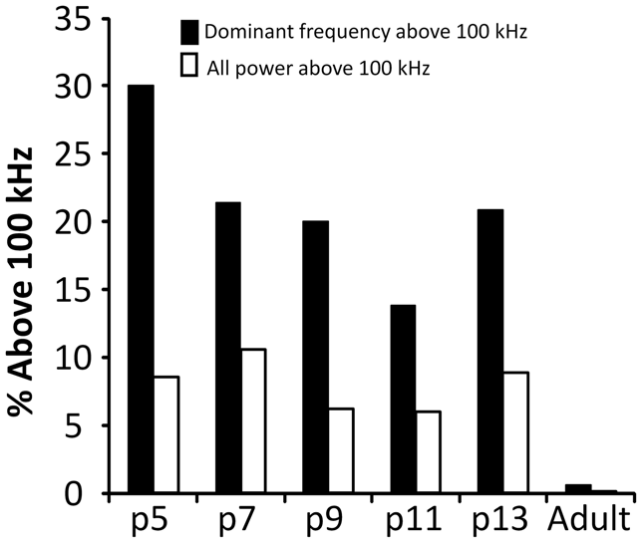
Syllables with dominant frequencies above 100 kHz are common in pups but not adults.

Surprisingly, the dominant frequencies of many pup syllables were above 100 kHz, the proposed upper limit of adult mouse hearing [24]. These frequencies are also above the range of mouse pup hearing; pups are deaf until p10 [6] and cannot hear frequencies above 50 kHz until after p14 [6], [7]. At p5, 30.1% (946/3145) of syllables had dominant frequencies above 100 kHz. This was still high at p11, where 13.8% (629/4560) of syllables had dominant frequencies above 100 kHz (Fig. 9). A smaller number of pup syllables, between 6% and 11% depending on the age group, had all of their energy above 100 kHz. These completely inaudible syllables would be perceived by adults as silent intervals within the bout, changing its temporal properties. Syllables with dominant frequencies above 100 kHz were rare in adult animals, 0.6% (43/6963), and no adult syllables had energy exclusively above 100 kHz (Fig. 9).

#### Harmonic structure

**Figure 10:**
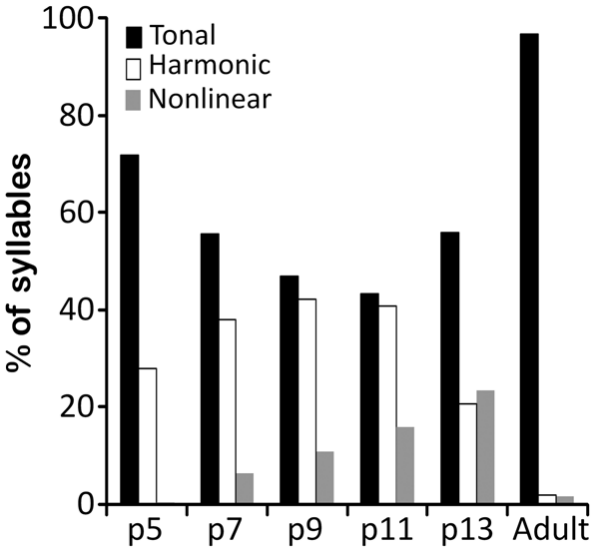
Harmonic and non-linear syllables are common in pups but not adults. With increasing pup age the proportion that had nonlinearities changed significantly (X^2^ (5, *N* = 2416) = 1417, *p*<0.001); increasing steadily developmentally. There were also significant changes in the proportions of harmonic syllables (X^2^ (5, *N* = 7672) = 1091, *p*<0.001). Adult mouse syllables were almost always tonal, the proportions of tonal syllables changed significantly over development (X^2^ (5, N = 17915) = 1737, p<0.001).

Most pup syllables and nearly all adult syllables were tonal, characterized by a single harmonic element (Fig. 2, all syllables marked “A”). In contrast, some pup syllables had either additional harmonics or nonlinear components. Figures 1 and 2 display several examples of harmonic syllables, with a second harmonic in the 100–200 kHz range (e.g., Fig. 1, all p9 syllables; Fig. 2, all syllables marked “B”). The two main types of nonlinearities were subharmonics, composed of frequency components that are integer fractions of the fundamental frequency, and deterministic chaos, composed of broadband noisy components (see [25]) (Fig. 2, all syllables marked “C”). Across pup age, there were significant age-related changes in the proportions of these syllables (Fig. 10). In particular, nonlinear syllables became progressively more common, increasing from 0.3% in p5 animals to 23.5% in p13 animals. Very few adult syllables were harmonic (1.7%, 121/6815) or had nonlinearities (1.6%, 106/6815).

### Features of vocal bouts

Mouse syllables were produced in bouts that could be composed of either the same syllable or a combination of different syllables. A bout was defined to include at least 3 syllables produced successively with silent intervals less than 1569.8 ms. This criterion, based on our analysis of all intervals less than 5000 ms, for all ages, is 2 standard deviations above the mean (mean = 337.8, SD = 616.0). Using this criterion, we analyzed 23608 syllables from 1337 bouts. We examine temporal features of the bouts, then the sequential ordering of syllables within a bout.

#### Temporal features of bouts

We compared inter-call interval (the silent interval between syllables), bout duration, and number of syllables in a bout across ages. The number of syllables in a bout decreased as a function of age (F (5, 1337) = 19, p<0.001). At p5, syllable bouts were comprised of an average of 24 syllables (95% CI 19, 26), decreasing to 15 syllables per bout at age p13. Adult bouts comprised of fewer syllables per bout than those of any pup age group (mean = 11, [95% CI 9, 12], p<0.001). The mean inter-syllable interval decreased significantly and substantially as a function of age (F (5, 23608) = 232, p<0.001). Because the distributions of syllable intervals were skewed towards shorter intervals, we describe this change using median values. The median inter-syllable interval was longest at p5 (190.2 ms), decreasing at each successive pup age except p11 (median values: p7, 149.1 ms; p9, 93.4 ms; p11, 96.5 ms; p13; 84.8 ms). The intervals between adult syllables were substantially shorter than those of pups of every age (median value, 69.4 ms).

#### Bout structure: probability analysis

Bouts of syllables typically comprised 3–4 syllable types in both pups and adults (mean = 3.8, [95% CI 3.7, 3.9]). We examined whether the occurrence of syllables within bouts followed non-random patterns, and whether patterns changed over development. Note that some developmental change in patterns is expected even if syllables are randomly distributed within bouts, since different syllable types have greater probabilities of being produced by animals of different ages. To analyze whether non-random patterns occurred, we examined pairings of syllable types using a two-syllable model (e.g. Flat-to-Chevron, Chevron-to-Chevron etc.). This approach tested whether the number of occurrences of syllable pairings differed from the number predicted by a random model. Syllable combinations were examined for all bouts with more than 3 syllables (n = 1337 bouts). The two-syllable model showed that the sequence of syllables within a bout was not random (Χ^2^ (9, *N* = 2039) = 10511, *p*<0.001).

We next compared the probabilities of switching between syllable types within a bout of syllables, across ages. With increasing age, the probability that animals switched between syllable types increased steadily from 0.43 at p5 to 0.63 by p13 (see Fig. 4B). The oldest pups, at p13, were similar to adults in their high probabilities of switching between syllable types within a bout. The increased probability of switching back and forth between those syllable types resulted in a more complex sequence of syllables being produced by older animals.

The transition with the highest associated probability changed developmentally. At both p5 and p7 the most probable transition was from a 1 freq. step syllable to a flat syllable; at p9 and p11, this changed to transitions from a 2 freq. step syllable to a 1 freq. step syllable. At p13 the most common transition was from a 2 freq. step syllable to a chevron syllable. In adult animals the most probable transition was from a 1 freq. step syllable to an up-FM syllable.

Bouts were most likely at every age to begin with a flat syllable, even though this was not the most common syllable type produced at each age. Flat syllables made up only 26% of adult syllables, yet the probability of a flat syllable being produced at the start of a bout was higher, at 0.46. The same pattern was observed for pups of all ages; there was a probability of between 0.32 and 0.64 of a flat syllable occurring first in a bout. The most probable 10-syllable bouts are shown in Table 2. The first syllable was computed based on the probability of that syllable occurring first in a bout. The second syllable is the syllable type that had the greatest transitional probability of following the first syllable type. Each successive syllable was the syllable type that had the highest transitional probability of following the previous two syllables, based on third-order transitional probabilities.

**Table 2:** Most probable sequences of syllables in 10-syllable bouts at each age, based on third-order transitional probabilities.

	Syllable type at each sequential position									
	1	2	3	4	5	6	7	8	9	10
**p5**	Flat	Flat	Flat	Flat	Flat	Flat	Flat	Flat	Flat	Flat
**p7**	Flat	1F. step	Flat	Flat	Flat	Flat	Flat	Flat	Flat	Flat
**p9**	Flat	Chevron	Chevron	Chevron	Chevron	Chevron	Chevron	Chevron	Chevron	Chevron
**p11**	Flat	2F. step	2F. step	2F. step	2F. step	2F. step	2F. step	2F. step	2F. step	2F. step
**p13**	Flat	Flat	Flat	Flat	Flat	Flat	Flat	Flat	Flat	Flat
**Adult**	Flat	Flat	Up	Flat	Up	Flat	Up	Flat	Up	Flat

#### Bout structure: information theory analysis

The two-syllable model described in the preceding section showed that the sequence of syllables within a bout was not random. This shows that at every age tested there was some sequential structure, in which the preceding syllable had an effect on what syllable type was likely to follow. We used information theory to compare the level of sequential organization across ages at the two-, three- and four-syllable levels. The overall entropy reduced with increasing entropy level (Fig. 4C); the negative entropic slope is indicative of higher-order structure within the sequencing of syllables. The entropy reduced significantly between H_1_ (first order) and H_2_ (second order) at all pup ages, and in adults (Χ^2^ (7–10, *N*>3143)>2059, *p*<0.001). This supports the findings of the two-syllable model. The entropy also reduced significantly between H_2_ and H_3_ (second to third order) at all pup ages and in adults (Χ^2^ (49–100, *N*>3143)>1204.19, *p*<0.001), showing that at all ages the probability of a particular syllable type being produced is affected by the two preceding syllable types. There was a further reduction of entropy between H_3_ and H_4_, this difference was not significant. Thus, at each age, bouts appear to be organized up to at least the three-syllable level.

A comparison of the degree of entropic change between H_1_ and H_3_ across development revealed an increase in the level of sequential structure. There was no difference in entropic change between p5 and p11 pups, but there was a significant difference between p11 and p13 pups. We also compared the overall difference in entropic change between pups younger than p13 and adults, and between p13 pups and adults; adults showed a greater degree of higher-order structure than pups of any age.

There was an increasing degree of higher-order structure as pups developed and from pups to adults. This was evident at both the two-syllable sequence level, H_1_ to H_2_, and the three-syllable sequence level, H_2_ to H_3_. There was a significantly larger reduction in entropy between H_1_ and H_2_ in p13 pups than in p5 pups (Χ^2^ (64) = 499, p<0.001), and between p13 pups and adults (Χ^2^ (100) = 6256, p<0.001). This difference was not evident between p5 and p11 pups. There was also a significant reduction in entropy developmentally between H_2_ and H_3_, reflecting a greater degree of organization at the three-syllable level. The entropy change between H_2_ and H_3_ increased between p5 and p13 (Χ^2^ (576) = 1347, p<0.001), and from pups younger than p13 to adults (Χ^2^ (1100) = 1295, p<0.001). Adults had 0.40 bits more organization at the two-syllable level than p13 pups (Χ^2^ (64) = 5627, p<0.001), however adults had no greater organization at the three-syllable level than p13 pups.

### Virtual Mouse Vocal Organ

The preceding sections described many developmental changes in the vocalizations of mice. We used these developmental changes to implement a probabilistic Markov model in order to generate bouts of syllables that have features appropriate for animals of different ages (instructions, program and associated sound files in Program S1).

**Figure 11:**
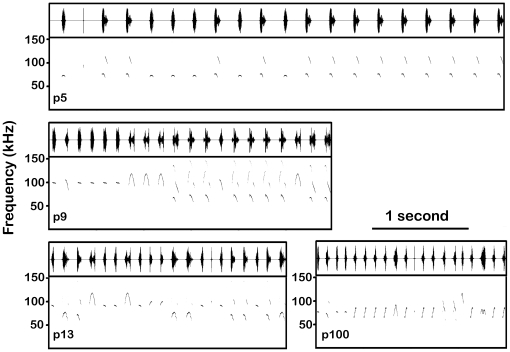
Spectrograms of syllable bouts generated by the Virtual Mouse Vocal Organ. Syllable bouts correspond to mice aged p5, p9, p13 and adult.

Figure 11 shows spectrograms of bouts of 20 syllables generated using the third order Markov model from p5, p9, p13, and adult mice. The bouts become dramatically shorter with development, as a result of overall decreases in both the duration of the individual syllables and the inter-syllable interval. The decrease in variability between the average frequencies of the individual syllable types is also evident. Note that some syllables, especially of younger pups, have significant or all energy above 100 kHz. These syllables would likely change the perceived temporal structure of bouts, as adult mice only hear frequencies up to approximately 100 kHz.

## Discussion

Our investigation into the developmental changes in mouse vocalizations combined several experimental features: examination of developmental changes for each of several syllable types, recording of spectral components in syllables above 100 kHz, and use of quantitative methods to examine the organization of mouse syllable sequences. This approach produced several key findings. 1) The CBA/CaJ mouse has 11 syllable types, 10 of which are produced by both pups and adults. The proportions of these syllable types vary developmentally. 2) Developmental changes in spectral and temporal features differ among syllable types. 3) The temporal characteristics of bouts changes with age, with older pups and adults having shorter intervals between syllables and fewer syllables within a bout. 4) The vocal repertoire becomes more diverse developmentally, with the overall complexity and higher-order structure of bouts increasing. Overall, this work characterizes the differences within mouse vocal communication that could be used by adult mice to determine the age of the vocalizing animal. The combination of age-dependent changes in the structure of vocalizations and the temporal sequencing of syllables was used to create age-appropriate bouts using the Virtual Mouse Vocal Organ.

### Developmental changes in syllable types

We observed all syllable types described in previous work in pups and adults [5], [13], [23], [26], [27]. In addition to the flat calls observed previously in CBA/CaJ pups [5], we recorded several syllable types with frequency transitions (see also Portfors [23]). Our study shows that each of the types observed in adults also occurs in pups, with the exception of the noisy syllable. Scattoni and colleagues [13] reported that at p8, the 2 freq. step was the most common syllable type produced by three commonly used mouse strains (30%, C57BL/6J; 41%, 129X1; and 43% FVB/NJ); this is in agreement with our finding that the 2 freq. step syllable was the most common at p9 in CBA/CaJ mice, representing 31% of syllables. These results suggest that even young mouse pups have well developed vocal production mechanisms which may be common across mouse strains.

The proportions of syllable types change over pup development. In p5 animals, most syllables were flat, 1 freq. step, or down-FM syllables. As pups age, they produced a greater variety of syllables, with generally more complex features. The changing proportions of different syllable types could be used as cues for mothers to distinguish pups on the basis of age. This change continues in juvenile mice, since syllable type distribution is quite different between p13 and adult animals.

### Developmental changes in spectro-temporal features of syllables

Our study corroborates results from previous studies showing decreases in the duration and frequency of adult syllables compared to those of pups [5], [13], [28], [29]. When we analyzed each syllable type separately, however, we found different patterns of developmental change. For some syllables, these patterns suggested a gradual change in the direction of the adult feature, but for other syllables or features, there was no such change.

#### Syllable frequency

Across all types of syllables, the frequency of syllables did not change in a systematic way with pup age. The average dominant frequency reduced steadily from p5 to p11, however after the onset of hearing, the dominant frequency of pup syllables returned to a value similar to p5 animals. This pattern was not reflective of all syllable types. Viewed individually, syllable types show different patterns of change in frequency. The complex syllable types had dominant frequencies that increased steadily with pup development, whereas the mean dominant frequency of the chevron syllable changed very little across development. The flat syllable showed a more complex pattern of change, as frequency formed a bimodal distribution. As development progressed, pups syllables more often fell within the higher frequency peak of the distribution, resulting in dominant frequencies that were above those of adult syllables.

Many calls from younger animals had the majority, if not all, of their energy at frequencies above 100 kHz, the proposed upper end of the mouse hearing range [24]. Pups of all ages produced many high frequency calls over 100 kHz, whereas adult animals did not produce any calls entirely above 100 kHz. It is possible that these calls reflect a lack of vocal control, though this seems unlikely because they were able to produce versions of each syllable type that are well within the mouse audiogram.

Puzzling in these patterns is the fact that many features change over the pup ages prior to hearing onset. Mouse pups are deaf until p10 [6] and cannot hear frequencies above 50 kHz until p14 [6], [7], thus they lack the acoustic feedback to recognize that these calls are inaudible. The changes we see could reflect increased motor control, although such explanations would need to account for changes in different directions for different types of syllables. Another factor that may underlie changing spectral features is selection by mothers. For these features, a more complete understanding of changes across development, including what occurs in juvenile animals, will help to assess the underlying mechanisms and functional significance.

#### Harmonic structure

Within pups, there was a steady increase in the proportion of syllables containing nonlinearities, from 1% at p5 to 23% at p13. However, in adults, there were virtually no syllables with non-linear spectral features. The increase in nonlinearities with pup age could relate to their functional properties in communication with mothers, or may be part of a developmental trajectory of the vocal organs. Our view below is that these nonlinearities are functionally important for older pups. However, any resolution of this issue requires an understanding of the developmental profile between p13 animals and adults.

Nonlinear components such as subharmonics, frequency jumps and deterministic chaos have been described in the vocalizations of several species, both vocal learners (zebra finch: [30]) and non-vocal learners (rhesus macaque:[25], pig: [31]; frog *Amolops torotus*: [32]). Several evolutionary benefits have been proposed for the use of nonlinearities within vocalizations. The acoustic variability they generate within calls is thought to enhance caller recognition in African wild dogs [33]. The formant frequency of vocalizations is used to ascertain the size of the caller in primates [34], [35], [36]. Primates potentially incorporate nonlinear phenomena so that their vocalizations seem to have a lower formant frequency, thus making the caller seem larger and audible at a greater distance [25].

We propose four plausible evolutionary reasons why producing non-linear pup vocalizations may be advantageous for mice. First, nonlinear components within syllables often make a sound lower frequency without reducing its overall sound level. In mice, smaller animals are often killed or neglected by the mother in favor of the larger ones [37], making it beneficial for the pup to appear larger. Second, broad band sounds are easier to locate than tonal sounds [38], thus the production of nonlinear calls may have developed to enhance the probability of the mother finding a pup when it has been removed from the nest. Third, nonlinear components may convey salience or emotional state that may be appropriate to isolation states in pups. Adult mice still produce nonlinear calls, though they are rare. It may be that nonlinear calls are a characteristic that adults associate with juvenile animals. Fourth, non linear components would generate mechanical distortion products in the cochlea at lower frequencies that would be audible by pups, thus pups may make many of these calls at p11 and p13 because they are self-audible. For example the non-linear 2 Freq. step syllable in Figure 2 would generate strong distortion product near 50 kHz and other, less strong, distortion products at lower frequencies (24–36 kHz). These are likely to be audible by pups above p10.

#### Temporal properties

A major difference between pup syllables and adult syllables is their duration [5], [13], [28], [29]. Pup syllables are on average 52 ms, while adult syllables are on average 29 ms. For some syllables, including the complex and the down-FM syllables, most of the decrease in duration occurs over pup development, with less or no change between p13 animals and adults. For other vocalizations (e.g., chevron and the 2 freq. step syllables), the major change occurs between p13 animals and adults. Still other syllables do not change substantially in duration over the times studied here (e.g., up-FM and short syllables). A major change across age, however, seems to be that older pups and adults produce an increasingly uniform distribution of syllable durations. For duration more than any other feature of syllable types, pups seem to be honing in on a particular duration across development, although how and when this occurs clearly varies for different syllable types.

#### Overall view

Viewed across the vocal repertoire of pups or adults, there are a variety of acoustic features of syllables, from frequency to duration to frequency-time properties, that permit adult animals to distinguish pups of different ages and pups from adults [5], [13]. We also recognize that pup syllables may be optimized to activate retrieval behavior by mothers, and so may undergo selection pressures and developmental trajectories that may be different than for adult syllables. To clarify these issues, it will be important to further characterize development of syllables in juveniles, and to test whether syllables develop similarly under different pup or adult hearing capabilities.

### Developmental changes in bout structure

There are age-related differences in the temporal patterns of bouts, with younger animals producing longer syllables with longer inter-syllable intervals. A greater repetition rate may convey information related to size of the caller. Syllables may be produced in bouts because sounds with sudden temporal breaks are easier to locate; arrival times at the two ears are easier to detect for sounds in a repetitious temporal pattern [38]. We found bouts were most likely, at every age, to start with a flat call. This is similar to the introductory notes characterized in zebra finch song, in which some notes are most likely to be produced first in a song [39].

#### Complexity of the vocal repertoire

If an animal's vocal repertoire is too unified (repetitious), then there is little communicative complexity, as a highly repetitious sequence of syllable types conveys only a little information. Conversely, if a repertoire is too diverse, or randomly distributed, the same message could be represented in multiple ways, resulting in less information being communicated by any one syllable type [16], [18]. The principle of Zipf's law [16] is that communication has an optimal balance between unification and diversification. The majority of human languages have an optimal balance within the proportions of word usage between unity and diversity with slopes of around −1[16]. Dolphin whistles become closer to this balance developmentally, achieving a value in adult dolphins that is close to human language, −0.95. Their developmental change in the slope is thought to reflect vocal learning. However age dependant differences in the Zipf slope have also been described in the squirrel monkey, an non-vocal learning species [16]. In both humans and bottlenose dolphin the repertoire begins as more diverse, becoming more repetitious over development. However, our data show changes that are more like the squirrel monkey, where the repertoire starts out overly repetitious and becomes more diverse across development.

The same proportion of syllable types can be composed within a bout of syllables with different levels of complexity. For example, the two sequences {A, A, A, A, B, B, B, B, C} and {A, B, C, B, B, A, A, B, A} have the same proportions of letters, however the overall complexity of the first sequence is less than the complexity of the second sequence. We rated the complexity of bouts of syllables by calculating the probability of transitions between syllable types occurring. As the animals aged, the probability of a switch between syllable types increased from 0.5 at p5 to 0.7 in p13 pups and adults, resulting in increasingly complex sequences. Finally, adult mouse vocalizations had significantly more higher-order sequential organization than p13 pup vocalizations, implying that the rules of the vocal system are still not fully developed by p13 and that more sequential learning may take place throughout adolescence.

#### Functional significance

Duration and frequency of sounds are critical for evoking pup retrieval behavior from the mother [40], [41]. Maternal mice respond with retrieval behavior to strings of pure tones at frequencies similar to pup calls, almost as well as they do to strings of complex pup calls [40]. If a pup need only produce flat tonal signals in order to evoke retrieval by its mother, why does it produce such complex strings of syllables?

Pups may increase the complexity of their bouts to make them distinct from those of littermates. In younger animals where the vocal repertoire is more repetitious, it is likely that the calls from one pup will be masked by the calls of other pups in the litter that are making similar sounds, making it harder for the mother to localize a specific pup. Pups may learn to produce more complex sequences to counter this masking and thus increase the likelihood of being localized, and retrieved, by their mother. A similar mechanism for the release of background masking has been reported in king penguin communication. Playback studies conducted in a quiet environment demonstrate that frequency of a syllable is the critical feature for retrieval. However, in an environment with background noise similar to the natural environment, the syllabic organization facilitates recognition [42].

Our results are consistent with vocal learning in mice, but do not provide sufficient supportive evidence. The changes we describe could result from vocal learning, developmental changes in motor control, or changes in the vocal tract. Vocal learning issues could be addressed by employing the analytic methods used here, comparing the vocalizations of normally hearing mice with those deafened prior to hearing onset and those raised by deafened dams.

### The virtual mouse vocal organ

We found several developmental changes within mouse syllables and sequences that could be used as acoustic cues for adults to determine the age of the caller. However, it is still unclear which of these cues are used by adult mice. We developed a virtual mouse vocal organ that creates appropriate syllable sequences based on age-related changes in several features that we measured: frequency, bandwidth, duration, inter-syllable interval, syllable probability and the sequential pattern (transitional probability).

This virtual mouse vocal organ consists of a third order Markovian probabilistic model of CBA/CaJ bouts of syllables. The model draws on the most typical version of each syllable type corresponding to each age tested and produces bouts of syllables in wav format. This can be used to generate well-controlled experimental stimuli for behavioral or neurophysiologic research (see attached MATLAB program).

## Supporting Information

Program S1 This compressed folder holds the 'Virtual Mouse Vocal Organ' program. The instructions for running the program are included along with the MATLAB code and a library of the most typical version of each syllable type produced at each age.
(RAR)
